# Correlates of Insulin Resistance in Nascent Metabolic Syndrome

**DOI:** 10.1177/11795514231168279

**Published:** 2023-04-19

**Authors:** Beverley Adams-Huet, Ishwarlal Jialal

**Affiliations:** 1 UT Southwestern Medical Center Dallas, Dallas, TX, USA; 2 Veterans Affairs Medical Center, Mather, CA, USA

**Keywords:** Metabolic syndrome, insulin resistance, inflammation, oxidative stress, fatty acids

## Abstract

**Background:**

Metabolic Syndrome (MetS), a major global problem, is a cluster of cardio-metabolic risk factors that predisposes to both type 2 diabetes mellitus (T2DM) and premature atherosclerotic cardiovascular disease (ASCVD). Insulin resistance is a major underpinning of MetS.

**Objectives:**

We investigated the relationship between insulin resistance and biomarkers of inflammation, oxidative stress, free fatty acids (FFA) levels and adipokine dysregulation in a cohort of nascent MetS.

**Design:**

This was a cross-sectional study comparing patients with MetS with matched controls.

**Patients and Methods:**

Participants included 47 patients with MetS and 41 controls. Persons with diabetes, ASCVD, smoking and macro-inflammation were excluded. Fasting blood was obtained for both plasma and monocyte isolation. Homeostasis model assessment insulin resistance index (HOMA-IR) was calculated from fasting glucose and insulin levels.

**Results:**

The patients were insulin resistant as determined by a valid measure, HOMA-IR. HOMA-IR increased with increasing severity of MetS and correlated with cardio-metabolic features, hsCRP, FFA levels, and adipose tissue insulin resistance. Insulin resistance also correlated with biomarkers of oxidative stress and both circulating and cellular biomarkers of inflammation. Receiver operating Characteristic (ROC) curve analysis revealed that HOMA-IR was an excellent predictor of MetS with an area under the curve of 0.80.

**Conclusion:**

In our patients with nascent MetS we show that they have significant insulin resistance. Based on our findings, elevated FFA levels, oxidative stress and inflammation could contribute to the insulin resistance.

## Introduction

Metabolic Syndrome (MetS) is a constellation of cardio-metabolic risk factors that predisposes to both type 2 diabetes mellitus (T2DM) and premature atherosclerotic cardiovascular disease (ASCVD).^1,2^ Additionally, it is a growing and major global problem with no optimum treatment.

Insulin resistance has been advanced as a dominant underpinning to explain the pathophysiology of MetS, largely based on the seminal work of Reaven and his group.^3-6^ However chronic low-grade inflammation appears to also be an important pathogenic mechanism as cataloged recently.^7^ Whilst there is a paucity of original reports on the insulin resistance of nascent MetS, other investigators have examined the role of insulin resistance in MetS.^8-11^ In the present report we investigate the relationship between insulin resistance and biomarkers of inflammation, FFA levels, oxidative stress and adipokine dysregulation in a select cohort of nascent MetS without the confounding of T2DM, ASCVD, smoking, chronic inflammation, and hypolipidemic drug therapy.

## Patients and Methods

In a series of papers, findings in this cohort focusing on adipokine dysregulation, inflammation and oxidative stress have been reported.^12-20^ MetS participants (n = 47) and controls (n = 41) aged 21 to 69 years were recruited from Sacramento County, CA using the criteria of the Adult Treatment Panel III (ATP III) as described previously.^1,12-20^ MetS volunteers had to have at least 3 of the 5 cardio-metabolic features used as criteria.^1,2,13,14^ Exclusion criteria for healthy control subjects included current use of any blood pressure medications, elevated triglyceride levels (>200 mg/dL) and having 3 or more of the ATP III criteria. Other important exclusion criteria for all subjects which were determined by a screening questionnaire, clinical examination and baseline chemistries included diabetes defined by fasting blood glucose level >125 mg/dL and HbA1C > 6.4%, clinical ASCVD, acute or chronic inflammatory disorders, and history of smoking. Major medication exclusion criteria for subjects with MetS included anti-diabetic medications, anti-coagulants, steroids, oral contraceptive therapy, estrogen replacement therapy, anti-inflammatory drugs, statins as well as other lipid lowering agents and angiotensin 2 receptor blockers. Additionally, all participants in the study had a high-sensitive C-reactive protein (hsCRP) level <10.0 mg/L and a normal white cell count. The study was approved by the institutional review board at the University of California, Davis and informed consent was obtained from all participants.

Fasting blood samples were taken from participants after histories and physical examinations. The details of the different assays have been reported previously.^12-20^ Germane to this report the homeostasis model assessment insulin resistance index (HOMA-IR) was calculated from glucose and insulin levels as follows: Fasting plasma glucose (mmol/L) × fasting plasma insulin (mU/L) Please include equation as in accepted paper ÷ *by 22.5*.^21^ It is important to emphasize that HOMA-IR is a valid measure of insulin resistance and has correlated significantly with the euglycemic clamp and minimal model methods.^21^ It has also been used in multiple population studies.^21^ Adipose tissue insulin resistance was calculated as the product of FFA and fasting insulin levels as reported previously.^16^

SAS version 9.4 (SAS Institute, Cary, NC) was used for statistical analysis and significance was defined as a 2-sided *P*-value < .05. Results are expressed as median and interquartile range. The Wilcoxon Rank Sum test was used to compare age and metabolic characteristics between controls and MetS subjects. Trend analysis of HOMA-IR levels with increasing number of characteristics of MetS in subjects was evaluated using the Jonckheere-Terpstra test. After combining the control and MetS groups, Spearman rank correlation coefficients were determined to assess the association between HOMA-IR and relevant variables. Logistic regression models were used to compute Receiver Operating Characteristic (ROC) Area under the curve (AUC).

## Results

In previous reports biomarkers of inflammation, oxidative stress and dysregulation of adipokine biology have been detailed in these patients.12-19 In the present communication the focus was on those biomarkers that were significantly abnormal in those published studies focusing on their relationships with insulin resistance quantified by a valid measure, HOMA-IR. There were no significant differences in age and gender between the 2 groups. As shown in Table 1, all 5 features of MetS were significantly different compared to controls. The median HOMA-IR levels were 2.5-fold higher in patients with MetS. In addition, insulin levels, non-HDL-cholesterol, hsCRP and the TG: HDL-C ratio were significantly increased in patients with MetS. Furthermore, adipose tissue insulin resistance (Adipo-IR) was also significantly increased in patients with MetS..

**Table 1.** Salient characteristics of patients with MetS compared to controls.

Variable	Control n = 41		MetS n = 47		*P* value*
	Median	25th-75th percentile	Median	25th-75th percentile	
Gender F/M	n = 33/8		n = 38/9		1.0
HOMA IR	1.11	0.98-2.85	2.78	1.86-5.81	<.0001
Age (y)	47	41-56	53	46-58	.10
BMI, (kg/m)^2^	29	26-33	33.5	31-39	.0001
WC (cm)	89	81-102	103	97-116	<.0001
BPs (mmHg)	119	110-129	130	125-138	<.0001
BPd (mmHg)	72	68-79	82	75-89	.0001
Glucose(mg/dL)	88	85-93	100	92-106	<.0001
TG (mg/dL)	72	60-97	150	103-174	<.0001
HDL-C (mg/dL)	53	43-64	39	33-47	<.0001
Non-HDL-C (mg/dL)	134	116-159	156	148-177	.0003
hsCRP (mg/L)	1.3	0.5-2.8	4.4	1.7-5.7	<.0001
Adipo-IR (mmol/pmol)	20.1	6.8-29.4	66.6	53.5-101.2	<.0001
TG:HDL-C ratio	1.5	0.9-1.9	3.7	2.2-5.4	<.0001
FFA (mmol/L)	0.35	0.16-0.44	0.80	0.70-0.89	<.0001
Insulin (mU/L)	7.1	3.8-10.9	12.8	9.0-18.0	<.0001

Abbreviations: Adipo-IR, adipose tissue insulin resistance; FFA, free fatty acids.

* Wilcoxon Rank Sum test

In the controls, 17 females were pre-menopausal and 16 were post-menopausal. HOMA-IR was not significantly different between these 2 groups; medians of 1.1 and 1.1, *P* = .36. In patients with MetS, 15 females were pre-menopausal and 23 were post-menopausal, once again there were no significant differences in HOMA-IR; medians of 2.4 versus 2.8, *P* = .50. However in both subgroups patients with MetS had significantly higher HOMA-IR levels compare to controls: premenopausal, medians of 2.4 versus 1.1 respectively, *P* = .02 and post-menopausal, medians of 2.8 versus 1.0 respectively, *P* < .0001.

**Figure 1.**
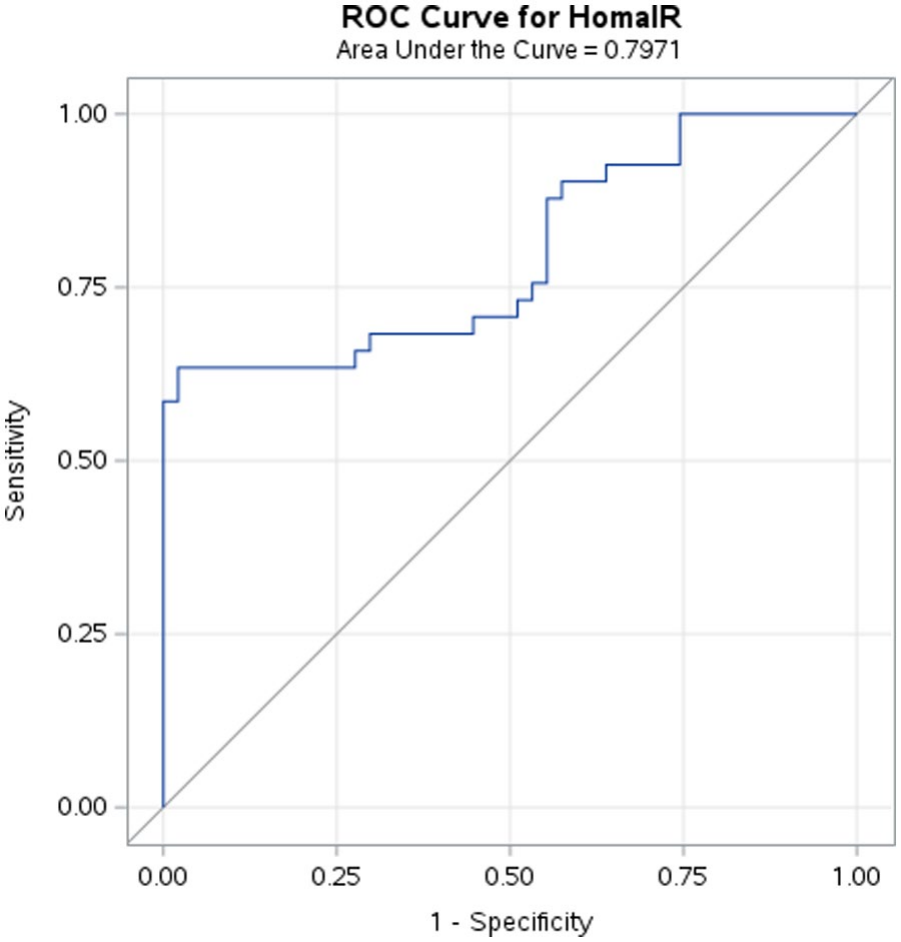
ROC-AUC of HOMA-IR predicting MetS.

ROC-AUC analyses of HOMA-IR in predicting MetS showed an excellent AUC of 0.80^22^ with confidence intervals (CI) of 0.70 to 0.89 as shown in Figure 1.

**Figure 2.**
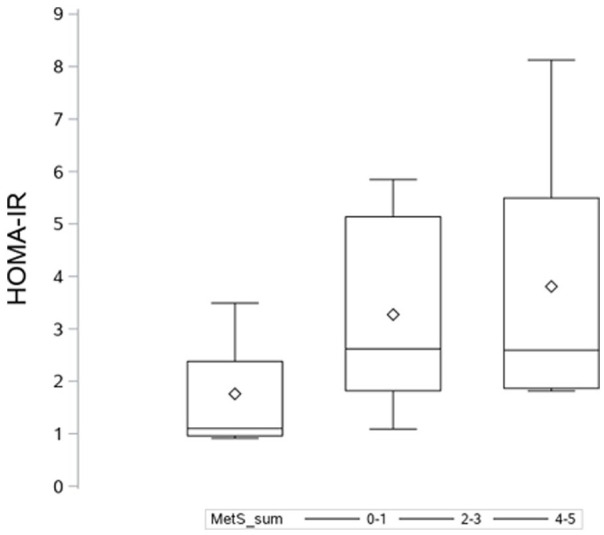
HOMA-IR levels with increased severity of MetS. The boundary of the box closest to zero indicates the 25th percentile, a line within the box marks the median, the diamond marks the mean, and the boundary of the box farthest from zero indicates the 75th percentile. Whiskers (error bars) above and below the box indicate the 90th and 10th percentiles.

As depicted in Figure 2, HOMA-IR increased with increased severity of MetS defined by number of cardio-metabolic features.

In Table 2 are shown relevant correlations with HOMA-IR. Systolic blood pressure, triglycerides, HDL-Cholesterol, FFA, hsCRP, TG: HDL-C ratio and Adipo-IR correlated significantly with HOMA-IR. There was a trend to significance with both waist circumference and plasma glucose levels (*P* = .05).

**Table 2.** Spearman Rank Correlation between HOMA-IR and relevant cardio-metabolic variables.

	Rho coefficient	*P*-values
Waist circumference (cm)	0.21	.05
BP-s (mmHg)	0.34	.001
BP-d (mmHg)	0.17	.12
Glucose (mg/dL)	0.21	.05
Triglycerides (mg/dL)	0.27	.01
HDL-C (mg/dL)	−0.23	.04
hsCRP (mg/L)	0.25	.02
FFA (mmol/L)	0.40	.02
TG: HDL-C Ratio	0.30	.004
ADIPO-IR (mmol/pmol)	0.86	<.0001

Whilst leptin and adiponectin did not correlate with HOMA-IR there was a significant correlation with chemerin levels (Table 3). Interleukin (IL)−6 but not IL-1 was significantly correlated with HOMA-IR.

**Table 3.** Correlations between HOMA-IR and biomarkers of inflammation, oxidative stress and adipokines.

Variable	*r*	*P*
Retinol binding protein-4	.22	.07
Leptin	.14	.27
Adiponectin	−0.17	.16
Chemerin	.40	.008
Interleukin-1β	.19	.07
Interleukin-6	.31	.004
Monocyte-Toll-like receptor-2	.18	.10
Monocyte-Toll-like receptor-4	.24	.03
Endotoxin	.48	.003
Soluble CD-14	.35	.008
Monocyte-NFkB activity	.37	.001
Monocyte-pP38MAPKinase activity	.40	.0005
Nitrotyrosine	.34	.03
Ox-LDL	.45	.001

Most interestingly HOMA-IR correlated significantly with monocyte cell surface toll like receptor 4(TLR-4) abundance and both monocyte (nuclear factor -Kappa-beta) NFkB activity and cytosolic phospho-P38- mitogen activated protein (MAP) Kinase activity. Also, HOMA-IR correlated significantly with plasma endotoxin and soluble CD14 levels.

In addition, HOMA-IR correlated with both downstream circulating biomarkers of oxidative stress; nitrotyrosine and ox-LDL.^14^

## Discussion

In this communication in patients with nascent MetS without the confounding of T2DM, ASCVD, macro-inflammation, smoking and hypolipidemic drug therapy, HOMA-IR establishes that they are insulin resistant and the insulin resistance increased with increasing severity of MetS. Furthermore, they also have increased adipose tissue insulin resistance that correlated very tightly (*r* = .85) with HOMA-IR. HOMA-IR correlated with a surrogate measure of insulin resistance, the ratio of TG: HDL-C as reported previously.^20^

There is a paucity of data on HOMA-IR and its inter-relationships in nascent MetS. Singh et al showed in male and female adolescent Indians with a mean age of 13.5 years that HOMA-IR was a reliable predictor of MetS.^8^ They did not report on adipokine dysregulation, inflammation or oxidative stress biomarkers but their study was free from confounders and HOMA-IR correlated with cardio-metabolic features of MetS. In Indian adults, Endukuru et al compared various surrogate markers of insulin resistance in controls and patients with MetS.^9^ Their study was confounded by 45% diabetics, 20% smokers and 27% on lipid lowering therapy. Despite these confounders they showed that the best surrogate for predicting MetS was HOMA-IR with a ROC-AUC of 0.85 (CI :0.78-0.90) and odds ratio of 2.24 (CI:1.6-3.1). Also the TG.glucose index (TyG) with a ROC-AUC of 0.84 and odds ratio of 1.51 performed well. In an Iranian population, Motamed et al showed that HOMA-IR was a reliable predictor of both MetS (ROC-AUC of 0.70 and 0.68 in men and women respectively) and non-alcoholic fatty liver disease.^10^ There was no mention of confounders except diabetes and no report of biomarkers of adipose tissue dysregulation, inflammation or oxidative stress like the present report. Son et al compared the HOMA-IR and TyG in a Korean population prospectively.^11^ They included patients with diabetes, smokers and lipid lowering therapy. They showed that TyG index was superior to HOMA-IR in predicting the prevalence of MetS: ROC-AUC of 0.84 and 0.68 respectively, *P* < .001. There was no mention of adipokines, biomarkers of oxidative stress or inflammation. The novelty of the present report is our attempt to elucidate the mechanisms of the increased insulin resistance in adults with nascent MetS by examining, adipose tissue dysregulation, inflammation and biomarkers of oxidative stress.

The most interesting correlations were with biomarkers of inflammation. In addition to hsCRP and IL-6 there were significant correlations with the pathogen recognition receptor of the innate immune response, monocyteTLR-4, and its classical ligand Endotoxin.^13^ FFA levels and soluble CD14, an accessory protein that primes endotoxin-TLR-4 activation also correlated significantly with HOMA-IR.^13,17,18^ In addition there were significant correlations with the downstream signal transduction pathways for TLR-4 including the master switch of inflammation, nuclear NFKB activity and cytosolic phospo-P38 MAPKinase activity.^9,15^ Many studies have reported a link between inflammation and insulin resistance and it has been argued based largely on studies in animal models that inflammation can presage and contribute to insulin resistance.^23-25^

In addition to inflammation, lipotoxicity (Elevated FFA levels, etc.) mitochondrial dysfunction and increased oxidative stress can contribute to insulin resistance.^23,25^ Plasma FFA levels correlated significantly with HOMA-IR in patients with nascent MetS and thus may contribute to the insulin resistance via activation of TLR4 and other potential mechanisms.^23-25^ Also 2 well accepted downstream footprints of increased oxidative stress, nitrotyrosine and Oxidized-LDL levels^14^ correlated with HOMA-IR suggesting that in nascent MetS also, oxidative stress could be an additional trigger for the increased insulin resistance.^23,25^

The data with respect to adipokine dysregulation were largely negative. There was a trend to a correlation with RBP-4 which promotes insulin resistance^7,12^ and significant correlations with chemerin and IL-6 with HOMA-IR. Chemerin, a chemoattractant for macrophages and dendritic cells is also an adipokine and appears to contribute to insulin resistance.^26^ In a prospective study, chemerin predicted the onset of T2DM over a period of 5.3 years.^27^ IL-6 is considered both a cytokine and adipokine^7,28^ and correlated with HOMA-IR. It is a crucial proximal step in the production of both the prototypic marker of inflammation, CRP, and fibrinogen.^7^ More importantly and germane to this report it promotes insulin resistance and T2DM.^25,29^

A major weakness of this report is the failure to assay sex hormones and determine their role in MetS. When this study was funded the major focus was on studying monocyte and adipose tissue biology and their roles in the inflammation of MetS. There is compelling data in men that testosterone deficiency predisposed to MetS and in females that estrogen deficiency predisposes to an increase risk of MetS.^30,31^ Furthermore studies have shown that sex hormone binding globulin (SHBG) also is an important determinant of MetS risk.^32,33^ In future studies this important area needs to be investigated in relationship to insulin resistance and inflammation and dysbiosis of gut microbiota.^30^

In conclusion, patients with nascent MetS without the confounding of T2DM, ASCVD, smoking, macro-inflammation and lipid therapy, have significant insulin resistance that increases with severity of MetS and is an excellent predictor of MetS. With respect to mechanistic insights, it appears based on the above findings that elevated FFA levels, oxidative stress and inflammation could be incriminated in the pathogenesis of the insulin resistance. However, given the cross-sectional nature of this report it cannot imply cause and effect. This can only be settled be prospective studies.

## Declarations

**Ethics approval and consent to participate:** Approved by UC Davis IRB: 200715074. All volunteers signed a written consent form prior to participation in the study.

**Consent for publication:** Not applicable.

**Availability of data and materials:** The data is available from the senior author for review on reasonable request.
